# Plant *in vitro* cultures: A promising and emerging technology for the feasible production of antidiabetic metabolites in *Caralluma tuberculata*

**DOI:** 10.3389/fendo.2022.1029942

**Published:** 2022-12-19

**Authors:** Amir Ali, Zia-ur-Rehman Mashwani, Ilyas Ahmad, Naveed Iqbal Raja, Sher Mohammad, Safir Ullah Khan

**Affiliations:** ^1^Department of Botany, PMAS, Arid Agriculture University, Rawalpindi, Pakistan; ^2^Biotechnology Laboratory, Agricultural Research Institute (ARI) Tarnab, Peshawar, Pakistan; ^3^Department of Cell Biology, Center for Research and Advanced Studies, National Polytechnic Institute of Mexico (CINVESTAV), Mexico City, Mexico

**Keywords:** secondary metabolites, diabetes, plant cultures, micropropagation, nanoelicitors

## Abstract

*Caralluma tuberculata*, a medicinal and edible plant of the genus *Caralluma*, belongs to the family Asclepiadaceae. Traditionally, its succulent stems are used as folk medicine for life-threatening diabetes mellitus (DM) disease. Its antidiabetic potential is ascribed to the presence of various secondary metabolites (e.g., pregnane glycosides, flavone glycosides, megastigmane glycosides, polyphenols, ferulic acid, quercetin, and bitter principles, among others) that act as effective and safe antidiabetic agents. The mechanisms of these bioactive secondary metabolites in *C. tuberculata* herbal medicine include lowering the blood glucose level, stimulating B cells of the pancreas to release more insulin, enhancing the sensitivity of the insulin receptor, inhibiting the action of glucagon and the hydrolysis of glycogen, and increasing the use of glucose in tissues and organ. However, overexploitation, alterations in natural environmental conditions, lower seed viability, and slow growth rate are responsible for the extinction of species from natural habitats, then becoming critically endangered species according to the International Union for Conservation of Nature Red List categories. Therefore, its limited availability does not meet the higher worldwide market demand of *C. tuberculata* as an antidiabetic drug. Thus, for its conservation and sustainable utilization, researchers across the globe are working on devising strategies to conserve and improve biomass along with the secondary metabolite profiles of *C. tuberculata* using *in vitro* approaches. The current review describes the recent progress on antidiabetic phytoconstituents, their cellular mechanisms, and their subsequent clinical outcomes in the drug discovery management of DM. Moreover, *in vitro* methods such as callus culture, micropropagation, and nano-elicitation strategies for conserving and producing bioactive secondary metabolites have been concisely reviewed and discussed.

## 1 Introduction

**Figure 1:**
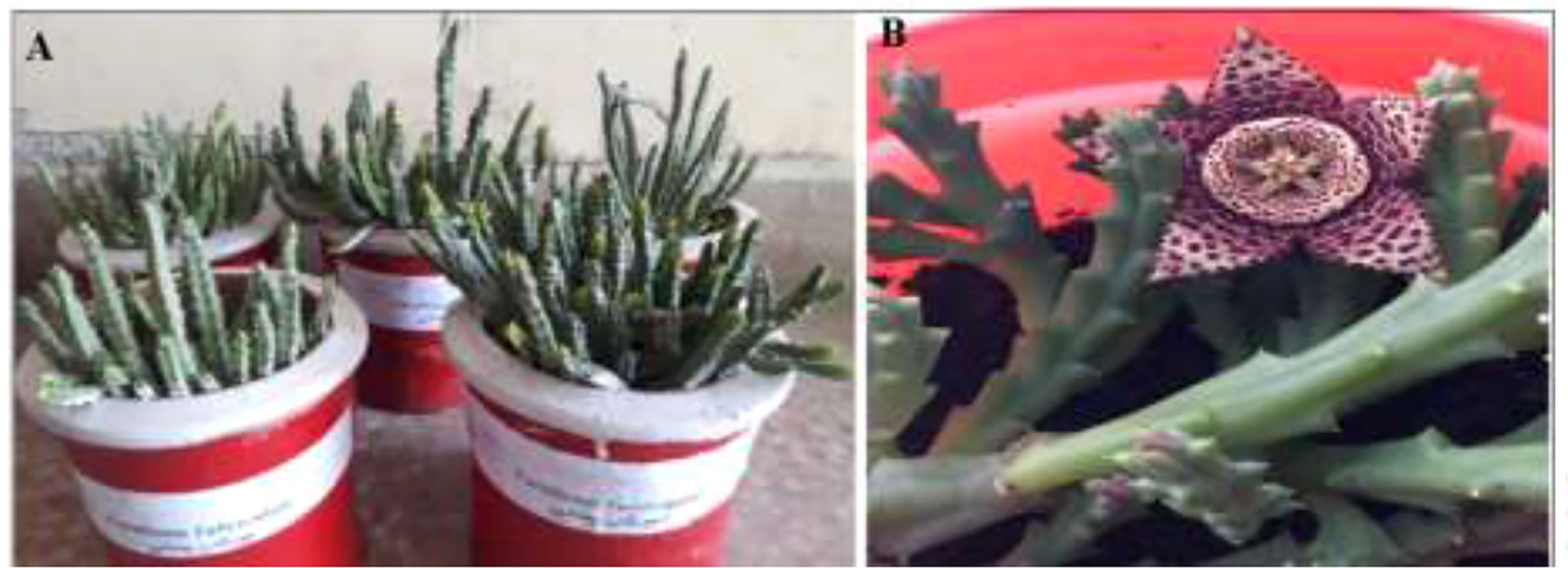
Antidiabetic herbal *Caralluma tuberculata* plant. **(A)** Potted *C tuberculata*. **(B)** *C tuberculata* flower.

*Caralluma tuberculata* is a high-value medicinal plant of the genus *Caralluma* and the family Asclepiadaceae. It has been previously cited for *Brussocia tuberculata* in the literature. *C. tuberculata* is a small, erect, fleshy, and leafless perennial herb with height from 45 cm to 1 m. The stem is angular, with soft spines in the notches. These spines have leaves that cover the angular stem (**Figure 1**). Inflorescence is a terminal cyme; flowers do not have the best scent, with color ranging from black, yellow, maroon, to dark brown (1). The fruit is long, narrow, erect, and smooth with a pointed apex. *C. tuberculata* can grow in dry, arid conditions with good aeration at temperatures above 10°C. However, it can grow well in greenhouse conditions. It is a native, critically endangered plant of Pakistan, but can be found in various dry, arid areas of Asia, Africa, and southeast Europe. The plant is consumed as a vegetable and is considered an antidiabetic agent due to its bitter taste. Therefore, destructive harvesting for ethnomedicinal purposes generally results in species extinction from its natural habitat (2). Traditional medicines from plant extracts have been confirmed to be effective in managing fatal diseases. Plants have extensive ethnomedicinal uses in the treatment of diabetes mellitus (DM) due to their secondary bioactive compounds that have various beneficial biological effects, thus potentially creating an effective and affordable multi-targeted treatment strategy (3).

According to documented results, the fleshy stem of *C. tuberculata* is a rich source of secondary metabolites such as terpenes, sterols, pregnane, glucoside, flavonoid, reducing sugar, tannin, and beta cyanine, which have solid antidiabetic potential (4). Furthermore, these bioactive compounds, particularly pregnane glycosides, could be valuable sources of natural antidiabetic drugs that could substitute conventional synthetic drugs that adversely impact human health (5). DM is one of the most severe complications associated with dysfunction, multi-organ failure, and uncontrolled levels of high blood sugar (6). The International Diabetes Federation reported that 389 million people are suffering from DM. Regular physical activities and a healthy diet can help control the risk of this fatal disease (7). Moreover, to prevent oxidative damage caused by DM, a rich natural source of nutrition is also helpful. Synthetic antidiabetic drugs are available for treatment, but have serious adverse effects. Therefore, there is an urgent need to identify novel and safe bioactive antidiabetic metabolite sources. *C. tuberculata* has been extensively used for diabetes treatment in urban and rural communities. Previous works on the biological activities of *Caralluma* spp., including their anti-hyperglycemic, anti-inflammatory, and protective properties, have been well explored. *C. tuberculata* is an extraordinary plant due to its being a high-value nutritious food for consumption and for its increasing pharmaceutical implications (8). Plants are important in pharmacological research and drug development, not only in the direct utilization of bioactive substances as therapeutic agents but also for use as starting materials for drug production or as models for pharmacologically active molecules. These metabolites are beneficial against infectious diseases with increased antibiotic resistance. Due to their abundant source and their ability to interact with various cellular processes, they can also serve as therapeutics, particularly in developing countries where access to healthcare is an issue (9). According to the WHO, 80% of the world’s population still uses herbal drugs as treatments against fatal diseases. In addition, about 25% of those currently employed in the pharmaceuticals industry extract medicinal compounds from aromatic and herbal drugs (10). The biological and medicinal significance of *C. tuberculata* has been attributed to glycosides. Glycosides are condensed products derived from a sugar and a non-sugar compound with a substituted or non-substituted ring structure. The main phytochemical ingredients in *Caralluma* are pregnane, flavone, megastigmane glycosides, saponin, and triterpenes. Generally, *C. tuberculata* is rich in glycosides, which could be useful in the treatment of DM (11). It also contains some other essential bioactive compounds such as cardiac glycosides, acylated steroidal glycosides, coumarin, emodins, anthocyanin, betacyanin, alkaloids, tannins, and several reducing sugars, essential oils, hydrocarbons, and other fatty acids (12). Phytochemicals, including polyphenols and flavonoids, have strong potential to scavenge free radicals and reduce oxidative damage, which are useful in the treatment of DM (13). Their anti-hyperglycemic effects due to the binding to glucose transporters competitively inhibit α-amylase and α-glucosidase. In addition, terpenoids, saponins, gallic acid, and ferulic acid could increase insulin secretion and accelerate the uptake and utilization of glucose, improving the performance of the pancreatic tissue and reducing intestinal absorption. The antidiabetic activity of these plants is thought to be mediated *via* various mechanisms, including stimulating insulin secretion from pancreatic B cells, increasing insulin binding to receptors, reducing insulin resistance, and improving glucose tolerance. Other modes of action include enhancing glucose metabolism, improving B-cell mass and function, and increasing plasma insulin, decreasing the levels of circulating blood glucose (14). The breakdown of restorative carbohydrates through gastrointestinal enzymatic reaction is responsible for the increase in blood sugar levels. α-Amylase is a hydrolytic enzyme that catalyzes the breakdown of 1,4-glycosidase bonds in starch, glucose, and in a variety of oligosaccharides. Glucosidase is an enzyme hydrolase released with the cells lining the striated boundaries of the epithelial tissue in the small intestine. It causes postprandial hyperglycemia by catalyzing the hydrolytic degradation of oligosaccharides into absorbable monosaccharides (15). One of the most common ways to lower the levels of postprandial blood sugar is to suppress the levels of gastrointestinal enzymes using secondary metabolites from botanicals. Alkaloids need to connect to the selective or non-competent sites of the enzymes involved in this process, preventing the formation of an enzyme–substrate complex, which decreases the actual enzymatic interest in the grand scheme of things. The protoberberine alkaloid palmatine inhibits both α-amylase and α-glucosidase. Therefore, it is necessary to explore the secondary metabolites of *C. tuberculata* that possess antioxidant activities and that are capable of inhibiting key enzymes such as α-amylase and α-glucosidase for the treatment of DM using traditional systems (15).

There are two primary conventional methods for the propagation of *C. tuberculata*: seed germination and stem cutting (11). However, these traditional techniques are still limited because of low seed viability. Moreover, due to the overexploitation of this critically endangered species, the collection of a lot of stems for vegetative propagation has become extremely challenging. Taken together, the insufficient plant growth and productivity of *C. tuberculata* hinders fulfilling the market demands at the commercial level. Therefore, plant biotechnology has gained popularity with regard to resolving the issues of conservation and inadequate production of *C. tuberculata*. In the field of plant biotechnology, *in vitro* approaches have shown promise in establishing plant cell cultures for the conservation and sustainable utilization of *C. tuberculata*. Moreover, *in vitro* plant culture methods that provide aseptic growth conditions ensure the conservation and bulk production of *C. tuberculata* materials with the production of sustainable bioactive antidiabetic metabolites compared to natural environmental growth conditions (16, 17). Owing to its tremendous industrial importance, researchers and scientists across the globe have been focusing on *C. tuberculata* to standardize the *in vitro* protocols for its conservation and sustainable plant biomass utilization with secondary metabolite production.

The current review is an endeavor to explore all the available information on the role of *C. tuberculata* as an antidiabetic agent. It also discusses the conservation strategies and the *in vitro* approaches for the sustainable utilization of *C. tuberculata*, including callus and cell suspension culture, micropropagation, and the metabolomic elucidation of the biosynthetic pathways and secondary metabolites.

## 2 Diabetes mellitus

**Figure 2:**
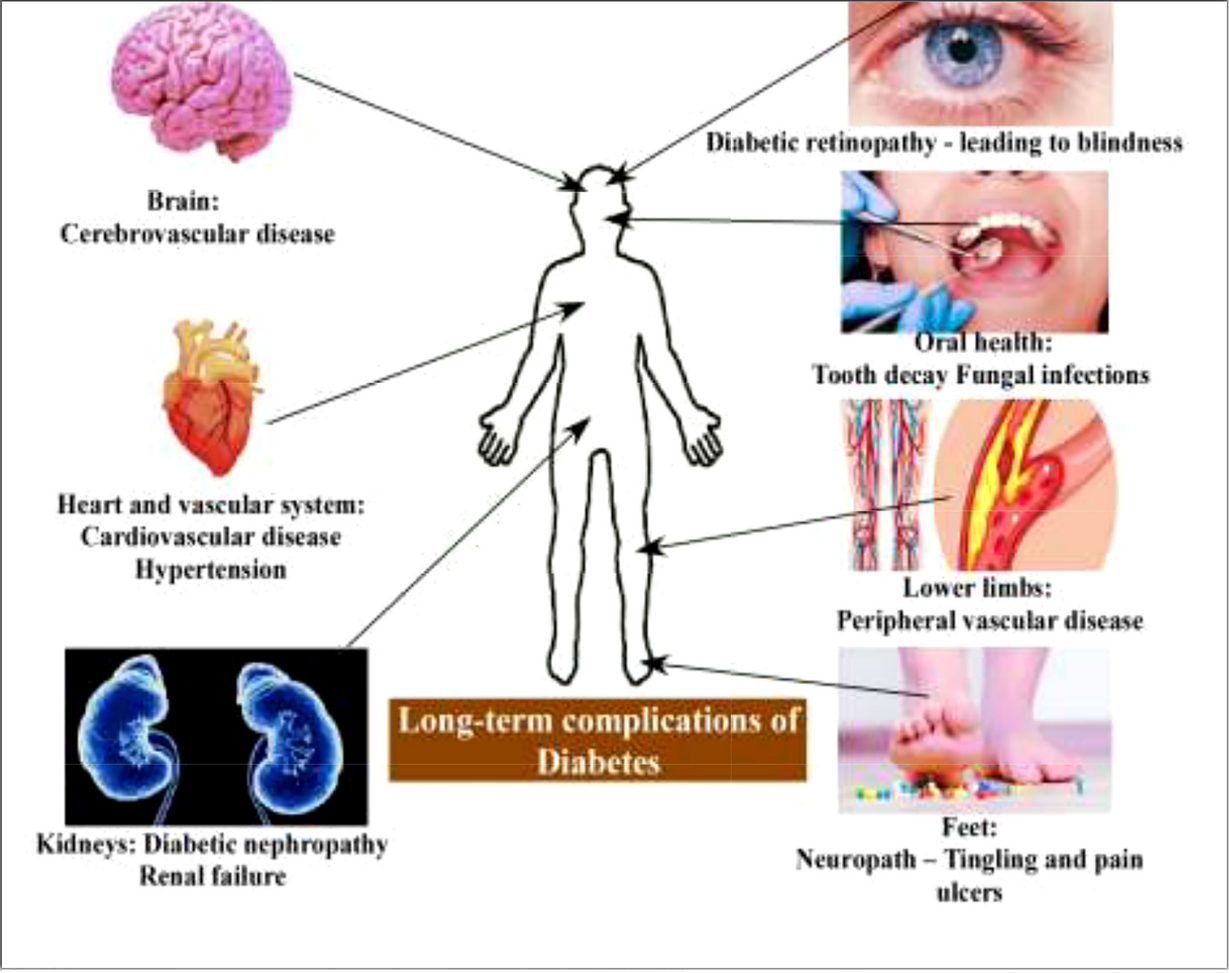
Long-term complications of diabetes (19).

**Figure 3:**
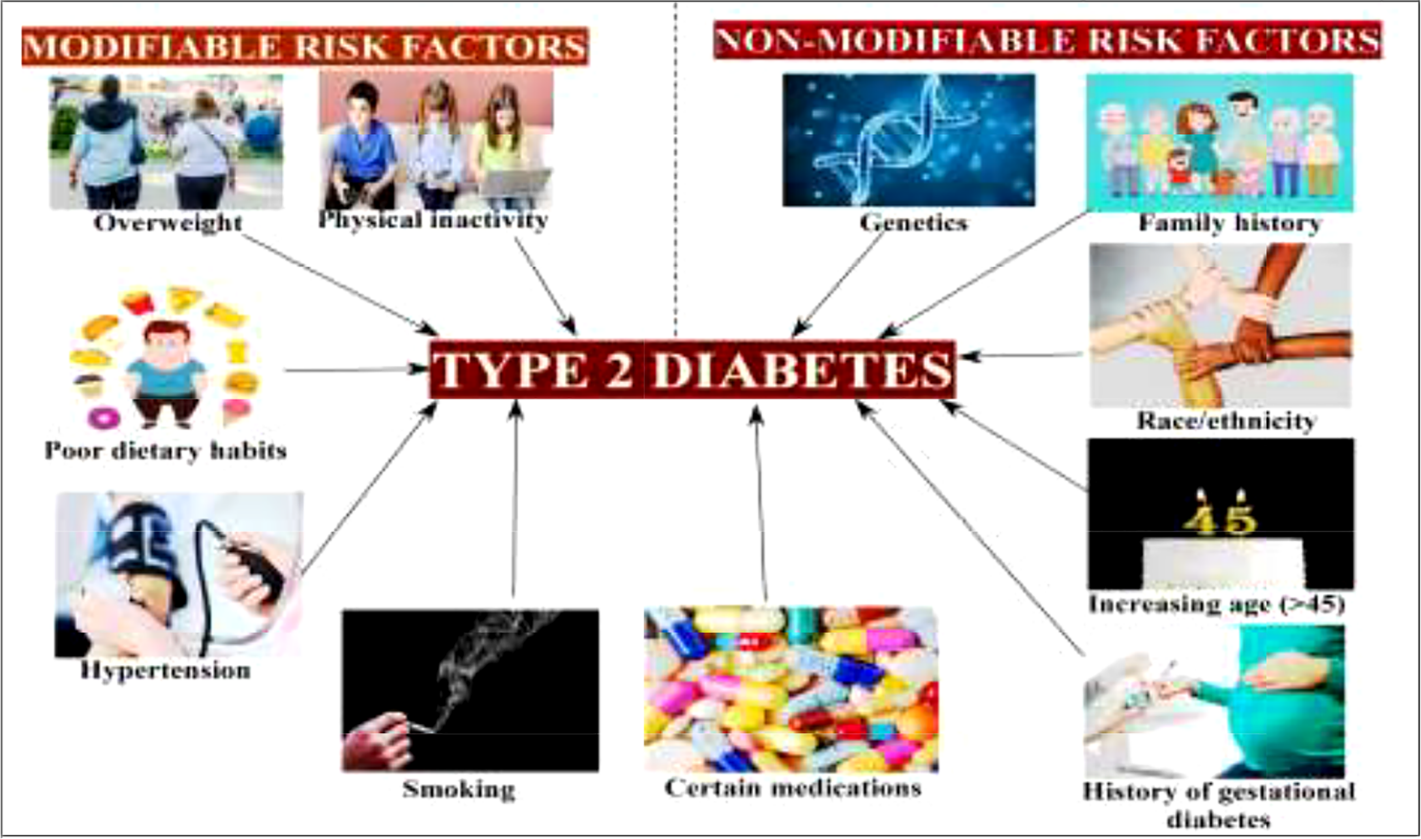
Modifiable and non-modifiable risk factors of type 2 diabetes mellitus (19).

DM is a metabolic disorder that prevents the body from adequately utilizing energy from consumed food. To better understand DM, it is necessary to have knowledge of the metabolic process by which the body uses energy from food. Much of the food consumed is broken down into a more straightforward form called glucose, which provides energy to the body. The blood vessels are the main sugar transportation routes because sugar itself cannot move into the cells. The most critical organ behind the stomach is the pancreas, which produces insulin hormone that acts as a carrier and lets sugar into cells (18). Therefore, without insulin hormone synthesis, sugar cannot enter the body’s cells and thus cause high levels of blood sugar, a condition called hyperglycemia. In addition, insulin deficiency can lead to the overproduction of reactive oxygen species (ROS), which cause B-cell dysfunction and other secondary complications (e.g., frequent urination, increased thirst, hunger, heart disease, kidney failure, foot ulcer, and eye damage) (**Figures 2**, **3**). According to the mechanistic approach, hyperglycemia activates various pathways, such as AGE/RAGE (advanced glycation end products and their receptor) formation, the protein kinase C (PKC) pathway, and the polyol and hexosamine pathway, and leads to ROS generation. The overproduction of ROS causes oxidative stress and inflammation, further activating the apoptosis pathway. In such conditions, they induce endothelium and vascular dysfuction by reducing the bioavailability of nitric oxide (NO). This results in the inhibition of insulin synthesis and consequently causes diabetes disease **Figure 5** (20, 21).

Therefore, there is an urgent need for novel therapies that do not have undesirable side effects for the management and treatment of diabetes. Recently, researchers and scientists have focused on a number of valuable highly medicinal plants for the design of effective strategies to discover novel therapeutic agents without any adverse effects (22). Conventionally established treatments for diabetes can cause some serious side effects. For instance, the most widely used antidiabetic drugs such as sulfonylureas and thiazolidinedione cause an increase in body weight. Moreover, biguanide and α-glucosidase therapy could negatively affect the gastrointestinal tract (23). As useful alternative sources, various medicinal plants have been recognized as antidiabetic agents through the separation and identification of novel bioactive secondary metabolites.

## 3 Use of the genus *Caralluma* as an antidiabetic agent

Naturally, plants produce potent secondary metabolites (e.g., terpenes, sterols, pregnane, glucosides, flavonoids, reducing sugar, tannin, and beta-cyanine) that are not only essential for growth and development but also serve as important novel medicines. There are new advanced strategies for the design of novel medicine for the treatment of diabetes based on ethnopharmacological and traditional knowledge of medicinal plants with identified secondary products (24, 25).

**Figure 4:**
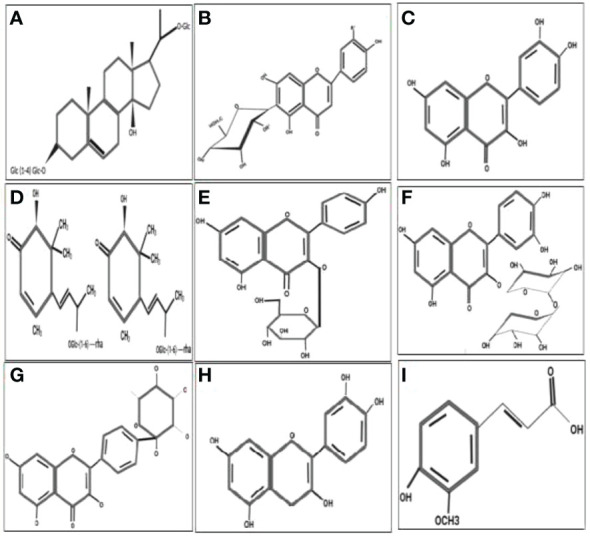
Molecular structure of the antidiabetic metabolites of *C aralluma tuberculata*. **(A)** Pregnane glycosides. **(B)** Flavone glycosides. **(C)** Quercetin. **(D)** Megastigmane glycosides. **(E)** Kaempferol 3-β-d-glucopyranoside. **(F)** Rutin. **(G)** Kaempferol-4′-*O*-alpha-l-rhamnopyranoside. **(H)** Catechin. **(I)** Ferulic acid.

Traditionally, herbaceous *Caralluma* species have been extensively used as folk medicine for hundreds of years to treat life-threatening DM. A lot of previous reports have indicated that the genus *Caralluma* offers a wide range of potent secondary compounds that can serve as effective and safe antidiabetic drugs (**Table 1**, **Figure 4**). Herbs from this genus are well-known medicinal plants due to the presence of two important compounds: pregnane glycosides and aglycone steroids (30). The literature survey showed that various parts (e.g., roots and stems) of *Caralluma* plants contain high-molecular-weight pregnane glycoside. The study by Abdel-Sattar et al. (26) investigated the potential impact of pregnane glycosides extracted from *C. quadarnula* on the glucose metabolism in the liver. The authors reported that pregnane glycosides enhanced the fasting serum glucose level, the glycated hemoglobin rate, lipid profile, and the serum insulin level (26).

**Table 1:** Antidiabetic compounds in the genus *Caralluma* and their modes of action.

Caralluma species	Antidiabetic compound	Mode of action	Reference
*Caralluma quadarnula*	Pregnane glycosides	Enhanced the fasting serum glucose level, serum insulin level, glycated hemoglobin rate, and lipid profile and accelerated the carbohydrate metabolic enzyme activities and the gene expression of glucokinase	(26)
*Caralluma tuberculata*	Flavone glycosides	Protected B cells from damage, improved their proliferation rate, and preserved insulin signaling through stimulating insulin secretion	(8)
*Caralluma umbellate*	Flavone glycosides	Elicited an increase the glucokinase level and promoted glycogen synthesis	(8)
*Caralluma arabica*	Kaempferol 3-*O*-beta-d glycoyranoside 4′-*O*-alpha-l-rhamnopyranoside	Reduced the glucose-6-phosphate and phosphoenolpyruvate carboxykinase gene expression and prevented glycogenolysis	(27)
*Caralluma nevegensis*	Flavone glycosides	Reduced glucose-6-phosphate and prevented glycogenolysis	(4)
*Caralluma europea*	Catechin, quercetin, rutin, ferulic acid, and hesperidin	Responsible for the free radical scavenging activity; activated all of the non-enzymatic (polyphenols) and enzymatic antioxidants (SOD, POD, CAT, and APx); improved the blood glucose level, total cholesterol, and serum glycerides; improved the lipid profile in high-fat diet rats; activated the liver enzymes; and reduced the fasting blood glucose level	(28, 29)

SOD, superoxide dismutase; POD, peroxide dismutase; CAT, catalase; APx, ascorbate peroxidase.

Furthermore, tremendous acceleration of the carbohydrate metabolic enzyme activities and the gene expression of glucokinase was also noted. The conclusion from this animal trial validated the therapeutic potential of pregnane glycosides in diabetes. The proposed mechanism for the hypoglycemic activity of pregnane glycosides might help reduce the blood glucose level through the absorption of intestinal glucose and stimulation of the release of the insulin hormone from the pancreas. According to Abdel-Sattar et al. (26), pregnane glycosides remain on the surface of the small intestine for a short period to absorb glucose, hence controlling the enhancement of sugar levels in the blood. On the other hand, pregnane glycosides are metabolized through the production of progesterone. Progesterone is an essential hormone that plays a primary role in controlling hyperglycemia, which shows dysfunction of pancreatic B cells. In addition, progesterone receptors significantly activate NO synthesis in the liver cells, improving insulin production (31).

Moreover, the insulin levels could be enhanced by the reduction of a glucogenic enzyme. The reduction of the activity of this enzyme can decrease the glucose levels in the blood (32). In addition to pregnane, flavone glycosides and other flavonoid ingredients were also evaluated in *C. tuberculata* and *Caralluma umbellate.* Flavonoids are a large group consisting of more than 6,000 compounds that are found in medicinal plants. A lot of flavone glycosides have great potential for antidiabetic activity, confirmed by the mode of action of these secondary metabolites particularly at the cellular level (33).

Flavone glycosides can protect B cells from damage, improving their proliferation rate and preserving their insulin signaling through stimulating the secretion of insulin. In addition, it has been proven that flavone glycosides are excellent elicitors that increase the glucokinase level, hence promoting the glycogen synthesis that ultimately reduces the gene expression of glucose-6-phosphate and phosphoenolpyruvate carboxykinase, resulting in the inhibition of glycogenolysis (34). The study by Kamil et al. (27) identified and separated a natural flavonoid known as kaempferol-3-*O*-beta-d-glucopyranoside 4′-*O*-alpha-l-rhamnopyranoside from *Caralluma arabica* using centrifugal countercurrent chromatography. Recent findings have provided evidence of the presence of two flavone glycosides in the methanolic extract of *Caralluma nevegensis* (4).

*Caralluma europea* is another popular species used for diabetic treatment. The phytochemical screening of *C. europea* has shown that the bitter extract contains several bioactive metabolites such as catechin, quercetin, rutin, ferulic acid, and hesperidin (35). Ferulic acid is the main active constituent of this herbal species related to its antidiabetic effect. Ferulic acid is a well-known non-phenolic phytochemical mainly responsible for the free radical scavenging activity. During stressed conditions, ferulic acid acts as a stimulator by activating the activities of all non-enzymatic (polyphenols) and enzymatic antioxidants such as superoxide dismutase (SOD), peroxide dismutase (POD), catalase (CAT), and ascorbate peroxidase (APx) (28). In a previous study (29), ferulic acid was used as an antidiabetic agent, which tremendously improved the blood glucose levels and other essential biochemical features, such as serum total cholesterol and triglycerides, in diabetic rats. Most research reports have acknowledged the significant role of ferulic acid in diabetes treatment as it improves the lipid profiles in rats fed a high-fat (HF) diet (36).

Another by-product abundantly present in *Caralluma* species is hesperidin, which controls hyperglycemia and hyperlipemia by scavenging free radicals (37). A clinical study showed that hesperidin supplementation significantly decreased the glucose levels in the blood by modifying the glucose-regulated enzymatic activity and normalizing the adiponectin and lipid levels. Moreover, numerous reports have also noted the influential role of hesperidin supplements in diabetes (38).

The rutin compound can be broadly isolated from *Caralluma* species. It was found to possess antioxidants in diabetes. Experimental reports demonstrated that diabetic mice treated with rutin showed significantly accelerated insulin levels, restoration of the glycogen content, and metabolic enzyme activities (39). Several mechanisms of the antidiabetic activity of rutin have been reported, including activation of the liver enzymes, reduction of the fasting blood glucose level, and oxidative stress intensity effectively (40). Wang et al. (41) speculated that the rutin compound could significantly improve body weight, decrease plasma glucose, improve myocardial dysfunction, and protect from oxidative stress, apoptosis, and inflammation in diabetic rat hearts. A recent investigation has demonstrated that rutin supplementation increased the brain-derived neurotrophic factor and nerve growth factor and enhanced Bcl-2 in the diabetic retina (42).

Quercetin is another secondary product that is naturally synthesized in *Caralluma* species. It is widely used as an antidiabetic agent to prevent and manage DM. The majority of studies have documented the mechanistic aspect of quercetin in diabetes. Supplementation with quercetin can reduce lipid peroxidation and accelerate the antioxidant enzymes SOD, POD, CAT, and APx (43). Kwon et al. (44) observed the impact of quercetin on Caco-2E intestinal cells and suggested that the transformation of fructose and glucose by glucose transporter 2 (GLUT2) was significantly inhibited by quercetin. Evidence from a study performed on diabetic rats declared that quercetin has an anti-inflammatory effect on adipose tissue and might be linked to the decline in body weight. However, the quercetin contents in the diet led to the recovery of proliferated cells in diabetic mice (45).

The mechanistic approach of the antidiabetic compounds in *C. tuberculata* involves pregnane glycosides, flavone glycosides, gallic acid, caffeic acid, and ferulic acid, among others, which enhance peripheral consumption and, therefore, reduce the plasma glucose levels without affecting the contents of serum insulin and liver glycogen. These derivatives have been reported to hinder macrophage infiltration and NF-κB activation; reduce the expressions of TNF-α, MCP-1, and plasminogen activator inhibitor type-1 (PAI-1); improve adipocyte differentiation; and lower the lipid profiles in experimental animal models. They stimulate the adenosine monophosphate-activated protein kinase (AMPK) that enhances glucose transporter (GLUT) translocation to the cell membrane, increases glucose transport into C2C12, inhibit the 3-hydroxy-3-methylglutaryl coenzyme A (HMG-CoA) reductase and acyl-coA cholesterol acyltransferase (ACAT) activities in rats fed a high-cholesterol diet, and stimulate the adiponectin secretion and AMPK phosphorylation, hence improving the insulin sensitivity compared to standard drugs. All these demonstrate the antidiabetic, anti-obesity, and anti-hypertension properties of these compounds (46) (**Figure 6**).

**Figure 5:**
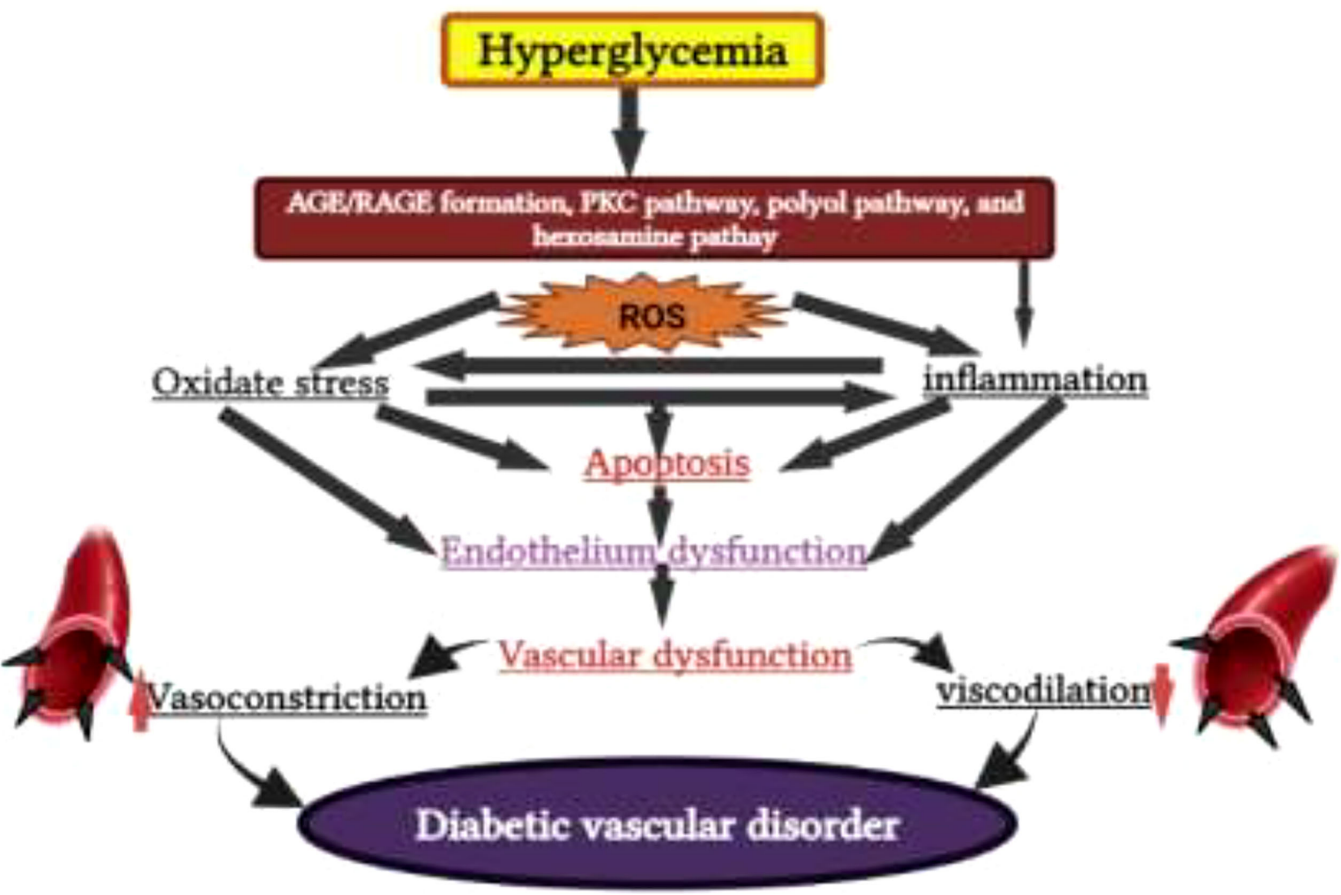
Hyperglycemia induces AGE/RAGE (advanced glycation end products and their receptor) formation, the protein kinase C (PKC) pathway, polyol pathway, and the hexosamine pathway and leads to reactive oxygen species (ROS) generation, which causes diabetes mellitus.

**Figure 6:**
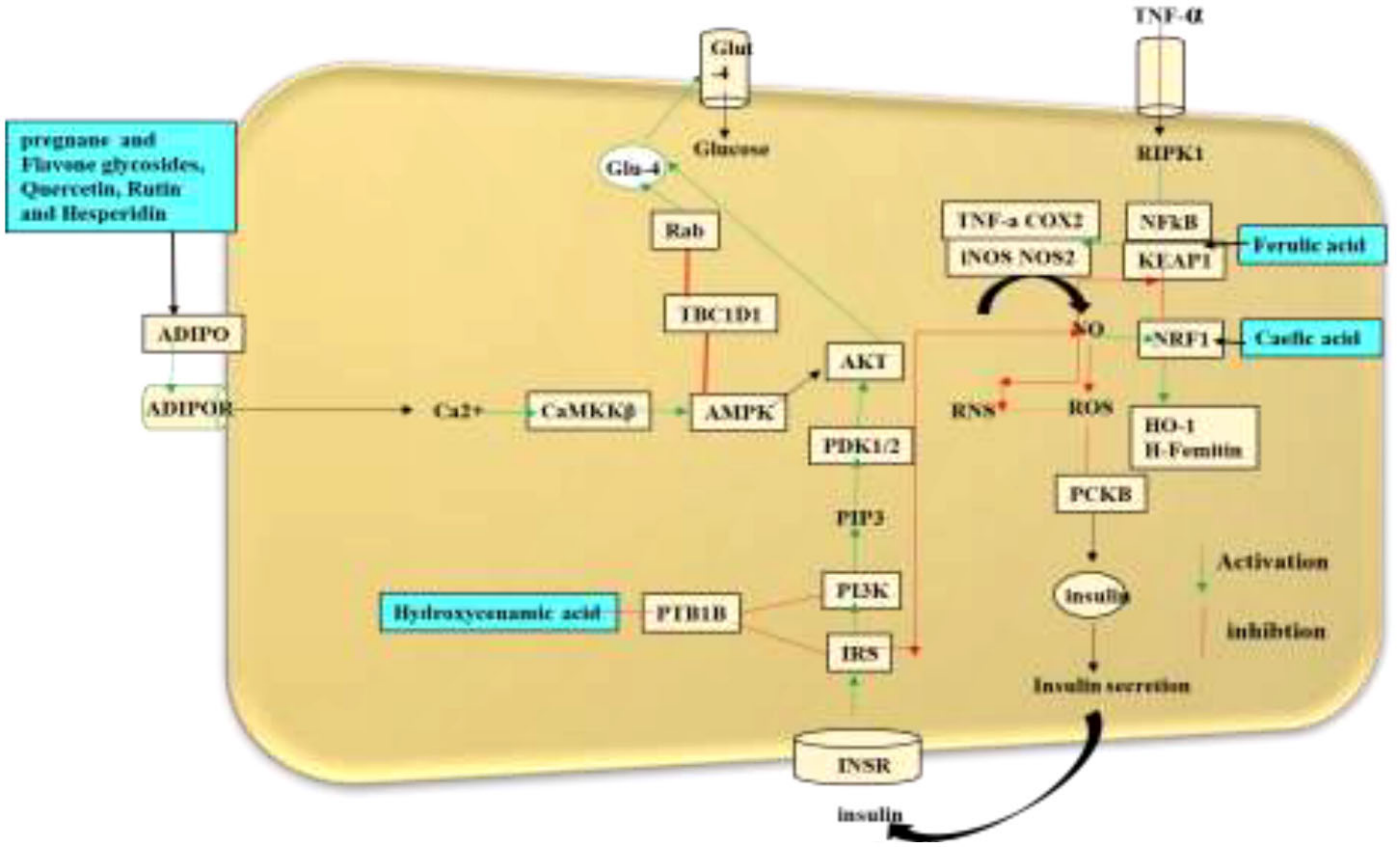
Schematic representation of the antidiabetic mechanisms of the natural compounds in *Caralluma tuberculata*.

## 4 Screening of Caralluma natural products for antidiabetic activity

*In vivo* and *in vitro* models have been extensively used in diabetes research to screen the potential of *Caralluma* species as antidiabetic agents (47). Their role as antidiabetic agents has been evaluated in terms of lowering the blood glucose levels in *in vivo* rodent models and insulin secretagogue activity (48), while *in vitro* models using muscle cells and adipocytes have been used to examine the insulin-like activity (49). Various experimental models can precisely reveal all the histopathological aspects and allows selecting one that is suitable to the aims of the study of diabetic disease (50). In DM research, mice and rats are the most commonly used models that provide insights on the metabolic signaling pathways, the development of insulin resistance, and hyperglycemia. Moreover, these mouse and rat models are also well acknowledged for genetic and physiological studies.

Therefore, *in vitro* and *in vivo* models are specifically beneficial for mechanism-based screening of the plant extracts and bioactive metabolites of *Caralluma* plants. Consequently, they are endorsed as the first step to assessing the antidiabetic capacity of these products. Evaluation of the insulin secretagogue activity, insulin-sensitizing potential, and enzyme inhibition are also common approaches in recent diabetes research. These can be performed using *in vivo* and *in vitro* models, which are less expensive, more effective, and do not have stringent ethical requirements (51).

**Figure 7:**
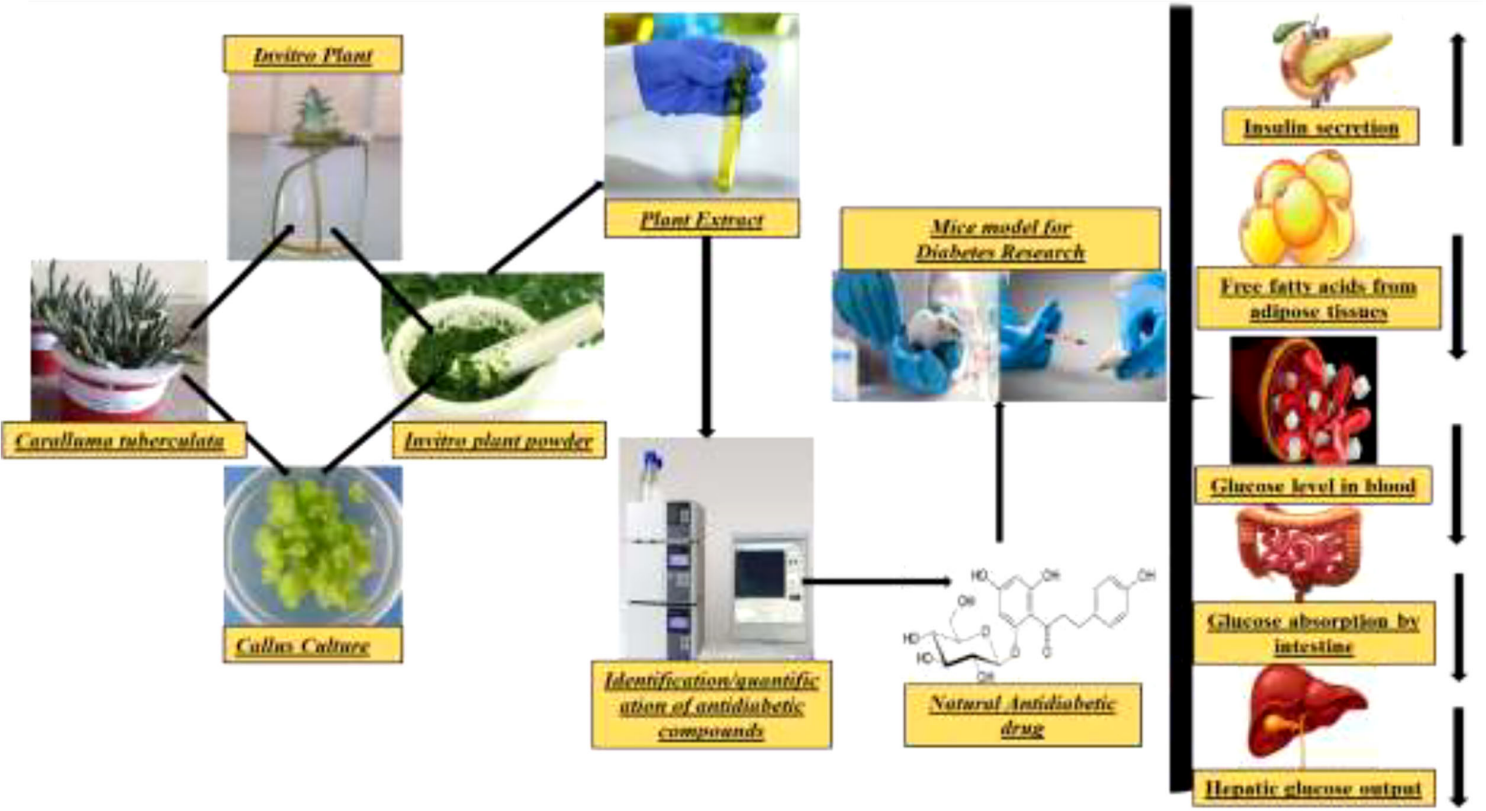
Graphical abstract of the review article.

Different extracts of *Caralluma* species have shown positive anti-hyperglycemic impacts and thus might be a favorable route in the management of diabetes. There are several antioxidant profiling studies on the extracts derived from *Caralluma* species considered as reducing agents that break the free radical chain reaction (i.e., free radical scavengers), thus displaying antidiabetic activity in different animal models (**Table 2**, **Figure 7**). For instance, Habibuddin et al. (52) examined the effect of the alcoholic extract of *Caralluma sinaica* on streptozotocin-induced diabetes in rabbits. The authors reported that augmentation with *C. sinaica* at various concentrations caused a significant reduction in the glucose level (*p* < 0.01) compared to clinically available glibenclamide drugs. All of these positive impacts support the use of this plant as an antidiabetic agent in the management of this fatal disease.

**Table 2:** Screening of *Caralluma* natural antidiabetic products using animal models.

Caralluma spp.	Plant extract	Animal model for clinical trials	Antidiabetic activity	Reference
*Caralluma sinaica*	Alcoholic extract	Streptozotocin-induced diabetic rabbits	Significant reduction in the glucose level	(52)
*Caralluma tuberculata*	Powder	Streptozotocin-induced diabetic rats	Antioxidant and anti-hyperglycemic activities	(53)
*Caralluma fimbriata*	Hydro-alcoholic extract	Wistar rats	Improved plasma glucose, insulin, leptin, and triglycerides in high-fat diet-induced rats	(54)
*Caralluma fimbriata*	Ethanolic extract	Alloxan-induced diabetic rats	Reduced blood glucose	(55)
*Caralluma tuberculata*	Extract given in capsule and cooking oil forms	Alloxan-induced diabetic rabbits	Significantly reduced the glucose level in blood	(56)
*Caralluma attenuate*	Alcoholic extract	Alloxan-induced diabetic rats	Reduced the blood glucose level	(57)
*Caralluma umballeta*	Ethanolic extract	Streptozotocin-induced rats	Decreased the blood glucose levels in diabetic rats	(58)
*Caralluma europea*	Crude Extract	*In vitro* model (baker’s yeast and rat intestinal α-glucosidase and α-amylase)	Highest inhibition of baker’s yeast α-glucosidase and improved the lipid profile	(59)
*Caralluma adscendens*	Methanolic extract	Alloxan-induced diabetic rats	Lowered the glucose concentration and improved glucose utilization in diabetic rats	(60)
*Caralluma fimbriata*	Methanolic extract	High-fat diet-induced diabetic rats	Significantly restored the glycogen rate in the muscles and liver and also the key enzymes of carbohydrate metabolism	(61)

Another study by Poodineh et al. (53) elucidated the antioxidant effects of *C. tuberculata* in streptozotocin-induced diabetic rats. This study administered different doses of *C. tuberculata* powder to diabetic rats for 7 weeks. The results showed that treatment with *C. tuberculata* powder demonstrated an ameliorative impact on blood glucose. Moreover, *C. tuberculata* plant materials also ameliorated the oxidative stress caused by diabetes in rats. Therefore, it appears that supplementation with an appropriate dose of *C. tuberculata* has both antioxidant and anti-hyperglycemic activities. Based on the results of several previous reports, we propose that *C. tuberculata* might be considered a preventive agent to protect tissues from oxidative stress during diabetes complications.

Sudhakara and his team investigated the beneficial effects of *Caralluma fimbriata* extract on insulin resistance and oxidative stress in Wistar rats. Their study justified that the hydroalcoholic extract of *C. fimbriata* had a solid capacity to improve the levels of plasma glucose, insulin, leptin, and triglycerides in rats given a HF diet. Therefore, we can conclude that administration of *C. fimbriata* extract could be valuable for suppressing HF diet-induced insulin resistance and oxidative stress (54).

Ashwini and his partners evaluated the antioxidant and antidiabetic activity of *C. fimbriata* in an alloxan-induced diabetic rat model. They successfully administered aqueous and ethanolic extracts of *C. fimbriata* for 1 week. The outcomes of this study described that the ethanolic extract exhibited more antidiabetic activity by reducing the blood glucose levels relative to the aqueous extract. These data on the antidiabetic effect of ethanolic extract validated the ethno-medicinal use of *C. fimbriata* as an effective antidiabetic herbal drug in the management of diabetes using folk medicine (55).

The study of Faisal et al. (56) indicated the antidiabetic potential of *C. tuberculata* extract in alloxan-induced diabetic rabbits. After 14 days of the induction of diabetes in rabbits, *Caralluma* extract was administered in capsule and cooking oil forms to assess hypoglycemic activity. The authors concluded treatment with the extract of *C. tuberculata* in the cooking oil form significantly reduced the glucose levels in the blood. This novel finding provides support for the use of *C. tuberculata* as an alternative treatment for DM.

Similarly, the hypoglycemic effect of different extracts (aqueous and alcoholic) of *Caralluma attenuata* was examined in alloxan-induced diabetic rats (57). The extracts showed a positive impact by reducing the blood glucose level in diabetic rats. Another report (58) examined the effect of an ethanolic extract isolated from *Caralluma umballeta* administered at a 400-mg/kg dose at different time intervals to streptozotocin-induced rats. Similarly to other study results, the extract showed healthy glycemic control by decreasing the blood glucose levels in diabetic rats. In Dra et al. (59), the authors explored the role of the phytochemical constituents extracted from C. *europea* in intestinal α-glucosidase and α-amylase in rats. Treatment with *C. europea* crude extract showed the highest inhibition of baker’s yeast α-glucosidase. These results indicated that the antidiabetic effect was due to the main compounds such as catechin hydrate and salicylic and caffeic acids, which may stimulate the remnant beta cells. In addition to the antidiabetic properties, the plant extracts showed robust antioxidant profiles and improved the lipid profiles of rats, which might have great potential application in diabetes treatment.

In 2009, Mali et al. (60) carried out an *in vivo* experiment on *Caralluma adscendens* to evaluate its hypoglycemic activity in alloxan-induced diabetic rats. Their results suggested that the extract of *C. adscendens* lowered the glucose concentration and improved glucose utilization in diabetic rats. In addition, there were five fractions of *C. europaea* prepared to elucidate the mechanism of the anti-hyperglycemic activity of this plant (62). The experimental study assessed the inhibitory effect of *C. europaea* on the α-glucosidase activity and intestinal glucose absorption. The results demonstrated that the extract of *C. europea* significantly inhibited the α-glucose activity and intestinal glucose absorption. This evidenced the many benefits of *C. europea* in the management of diabetes, which provides support for the use of this plant as an antidiabetic drug. Moreover, the study evaluated the modulatory impact of *C. fimbriata* extract by examining the enzymatic activity of carbohydrate metabolism and alterations in the glycogen content (liver and muscle) in HF diet-induced diabetic rats. This study observed that orally administered *C. fimbriata* extract significantly restored the glycogen rate in the muscles and liver, as well as the crucial enzymes of carbohydrate metabolism, in diabetic rats (61).

## 5 *In vitro* strategies to improve the production of biomass and secondary metabolites in C. tuberculata

Conventionally, *C. tuberculata* is propagated through vegetative and reproductive methods such as stem cutting and seed germination. The seeds of this plant are minute (4.9–7.0 mm), contain endosperm, and are easily dispersed by wind. However, the germination capacity and seedling growth are weak due to the small size of the seeds (63, 64). Therefore, the lower seed viability of *C. tuberculata* represents a huge hindrance to its commercial-scale production. Generally, propagation by seeds results in more heterogeneous plants. For this reason, propagation through stem cutting has been considered more valuable to achieving homogeneous *C. tuberculata* plantlets. For substantial root development, stem cuttings should be planted in sandy soil (65). However, natural environmental conditions and improper collection significantly influence plant survival, suppressing plantlet development. In addition, the limited availability of plantlets and the high labor input cause difficulties in vegetative propagation. Overall, traditional vegetative propagation strategies do not help in the conservation of and do not meet the market demands for highly threatened medicinal plants. On the other hand, *in vitro* strategies can conserve and produce considerable plant biomass to achieve medicinally active antidiabetic compounds. Thus, several approaches including *in vitro* micropropagation, callus culture, cell suspension culture, adventitious root culture, and nano-elicitation strategies have been standardized to improve plant biomass and the synthesis of bioactive metabolites at a feasible level in *C. tuberculata*.

### 5.1 Callus and cell cultures

*In vitro* plant cell culture approaches can play an indispensable role in the conservation and improvement of the secondary metabolite profiles of critically endangered *C. tuberculata* species. Recent innovations in plant cell culture techniques, such as the standardization of the culture medium to develop *in vitro* plantlets, the establishment of callus culture, and the application of suitable elicitors, have been widely employed to improve the production of hygienic germplasms and the fabrication of bioactive compounds at the industrial level (66). However, large-scale production of *C. tuberculata* is necessary for its industrial application. Significant obstacles are the low seed viability and the poor availability of vigorous stem cuttings. Therefore, *in vitro* plant culture is one of the approaches to achieving rapid and mass production of *C. tuberculata*.

In comparison to field-grown *C. tuberculata*, substantial amounts of secondary metabolites have been extracted from *plant* materials developed *in vitro*. Callus cultures can be considered a limitless source for the sustainable production of bioactive compounds under an aseptic environment. The establishment of callus culture in *C. tuberculata* relies on the explant type and the composition of nutrients and plant growth regulators (PGRs) in the growth media, the growth conditions, and germplasm conservation (67, 68). However, the culture conditions vary from species to species and need to be elaborated on in each case. Non-biological factors such as light, temperature, the pH of the medium, and the aeration of cultures influence the secondary metabolite biosynthesis pathways. Generally, callus cultures are sustained on a solid agar medium augmented with macro- and micronutrients (e.g., nitrogen, phosphorus, and potassium) and vitamins. Therefore, a low ammonium ion concentration in the growth medium stimulates the production of the secondary compounds relative to a high amount of ammonium, which inhibits the synthesis mechanism.

**Figure 8:**
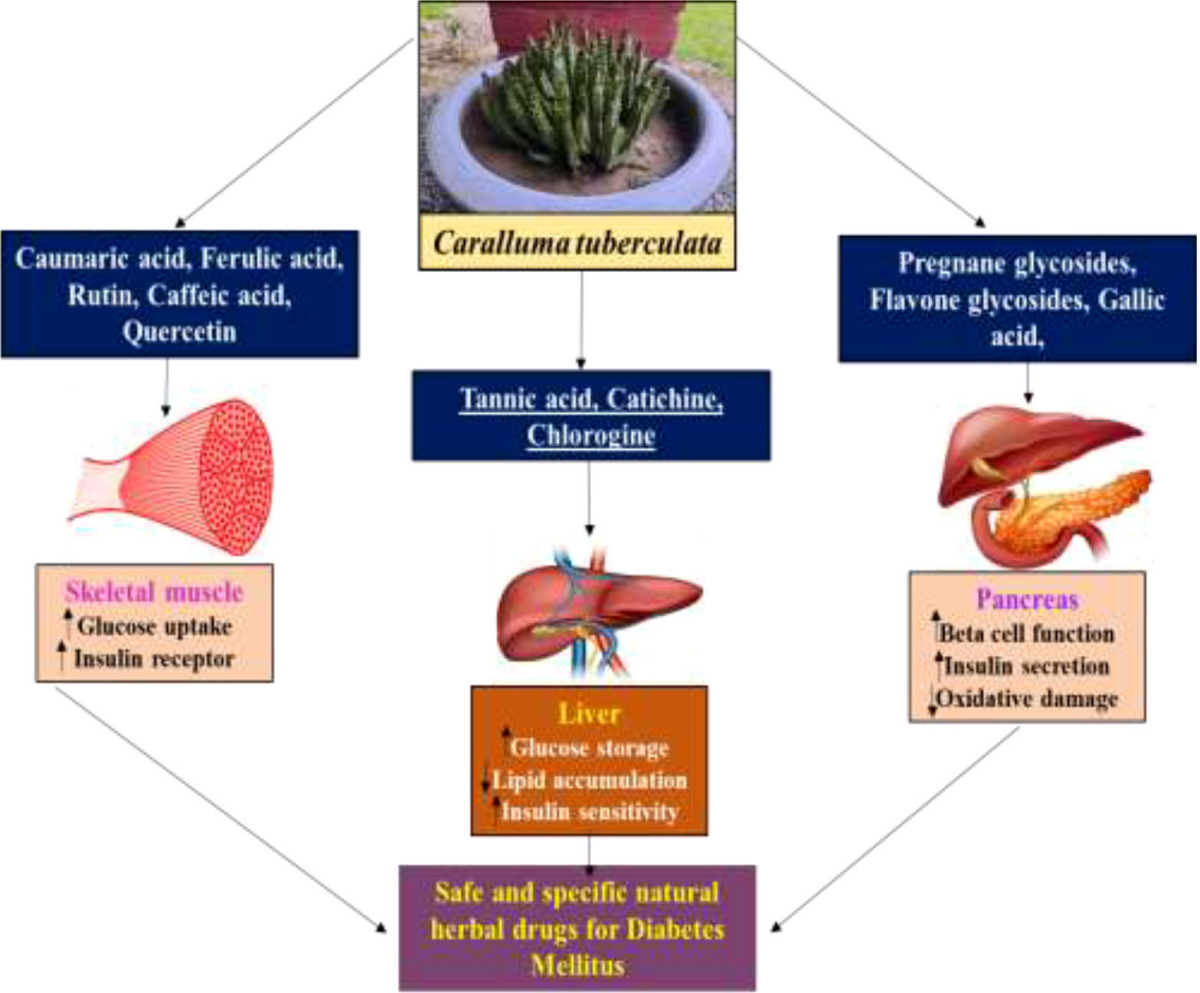
*Caralluma tuberculata* as a natural antidiabetic herbal drug.

Similarly, maximum phosphate levels normally regulate the cell growth and primary metabolism, while minimum phosphate levels are favorable for secondary metabolite formation (66). The notable advantages of the callus culture technique compared to conventional whole plant cultivation include the following: i) the callus material has the potential to sustain rapid cell division for an extended period and, hence, is suitable for the accumulation of potent secondary metabolites; i) the plant’s active compounds can be generated autonomously; ii) there is no threat to cultured cells (e.g., attacks by microorganisms or insects) due to the controlled environment; iii) the cells of any plant, even the rare or endangered ones, can be easily maintained (germplasm conservation) to produce their secondary metabolites; iv) it is a useful strategy for gene manipulation and genetic engineering; and v) robot-assisted regulation of secondary metabolite synthesis significantly reduces costs and improves productivity (**Figure 8**) (69–71).

Various reports have been published on the establishment of callus and cell culture protocols for *Caralluma* species. A study evaluating the effect of PGRs on callus induction and production (72) found that Murashige and Skoog (MS) media containing 2,4-dichlorophenoxyacetic acid (2,4-D) and 6-benzylaminopurine (BAP) resulted in maximum callus induction. Furthermore, the same hormonal combination in the growth media also accelerated the production of secondary metabolites in calli. This protocol can be utilized for germplasm conservation and large-scale production and elaborated the antioxidant value of this threatened plant species. A compact and greenish callus induced from the nodal segments of *C. fimbriata* cultured on MS media augmented with the plant hormones 2,4-D and BAP was reported by Rajaram et al. (73). In another study, the effective combination of 2,4-D and naphthaleneacetic acid (NAA) induced excellent frequency of callus in *C. adscendens* (74). Callus from internodal segments of the endangered *Caralluma stalagmifera* plant was induced successfully by inoculating explants in MS medium along with various ratios of auxins [i.e., 2,4-D, 2,45-trichlorophenoxyacetic acid (2,4,5-T), 2-(2,4,5-trichlorophenoxy)propionic acid (2,4,5-TP), NAA, indole-3-acetic acid (IAA), and indole-3-butyric acid (IBA)] and cytokinins [i.e., BAP, kinetin (Kn), 2-isopentenyl adenine (2-IP), and zeatin] either alone or in combination (75). This study recorded maximum callus induction rate (75%) on MS medium supplemented with 2,4-D (2.0 mg/l) alone.

#### 5.1.1 Micropropagation

One of the promising features of plant cells is their totipotency, which permits *in vitro* micropropagation under an aseptic environment with outstanding results relative to the conventional propagation methods, i.e., seed germination and stem cutting (76). Irrespective of the season and the year-round weather, thousands of plants can be produced from a single explant in a short period under a controlled environment. Similarly, a single explant can be multiplied into several thousand plants in a relatively short period and a small space under controlled conditions all year round (77). Moreover, critically endangered species are efficaciously conserved and grown with the use of the micropropagation technique because fewer plants are required for induction. Subsequently, *in vitro* propagated plants can be further multiplied at a larger scale.

Additionally, *in vitro* plant culture is the most efficient tool for improving the clonal and gametoclonal variants of different crops. This technology has great capability of producing high-yielding, disease-resistant, and stress-tolerant plants (78). The selection of a suitable range of PGRs in a media, the explant type, and the growth environment expressively influence the establishment and the development of medicinal plant cultures. In circumstances of adequate plant growth regulators, research reports have recognized that the addition of BA and NAA in MS media promoted the successful *in vitro* micropropagation of the pharmaceutically valuable *C. adscendens* species (79).

Ugraiah et al. (80) documented an efficient protocol for the conservation and rapid *in vitro* propagation of the critically endangered medicinal plant *Caralluma bhupenderiana* Sarkaria from nodal explants. This research study examined the physiological impact of various growth regulators (BAP, Kn, 2-IP, zeatin, IAA, and IBA) at different strengths in MS medium. The MS media augmented with BA produced maximum shoots, whereas a feasible number of roots per plant was achieved with half-strength MS medium along with NAA. Similarly, Karthik et al. (81) standardized a cost-effective and efficient procedure for the conservation and large-scale commercial production of *Caralluma diffusa* through *in vitro* organogenesis employing nodal explant culture media (MS) fortified with different concentrations of BAP, 2,4-D, NAA, and Kn. Positive response on shoot induction was recorded in nodal segments when cultured in MS medium containing BAP. Following root initiation, half-strength MS medium was supplied with the combination of IBA and NAA. This method can be productively utilized in long-term *in vitro* conservation and commercial-scale propagation of *C. diffusa*. Moreover, the micropropagation technique is suitable for conservation and rapid production and improves the antioxidant profile of *C. tuberculata*. A typical antioxidant profile describes the nutritional and pharmaceutical importance of the threatened *C. tuberculata* species.

#### 5.1.2 Nano-elicitation strategy for feasible biomass and secondary metabolite production in *in vitro* callus cultures of *C. tuberculata*

Plant derivatives of potent secondary bioactive products are a natural source of materials for the pharmaceutical, cosmetic, and food industries. A widely known technology, *in vitro* plant cell culture has been recommended worldwide as a promising alternative for the production of biomass and essential secondary products. However, this technique is still limited in producing biomass and important medicinal compounds to enhance the synthesis of plant secondary metabolites at the industrial scale for commercial purposes. Different approaches for *in vitro* culture have been optimized to accelerate the manufacturing of plant products (82). In this regard, scientific advancements in *in vitro* culture have proposed an elicitation strategy employing various elicitors such as melatonin, inositol triphosphate, jasmonate, ethylene, abscisic acid, and nanoparticles (NPs). These elicitors are involved in achieving feasible biomass production by triggering,—that is, they elicit—the defense machinery of plant cells to combat oxidative stress. The biotic or abiotic factors that activate the defense machinery of the plant are called elicitors (83). Elicitors interact with the cell membrane at the initial stage. Various receptors, such as plasma membrane-associated receptors, are then stimulated to elicit the bioactive compounds for defense. Subsequently, signal reception is followed by transduction, which consists of multiple phases including the reversible phosphorylation and dephosphorylation of the plasma membrane and cytosolic proteins; acceleration of Ca^2+^ in the cytosol; H^+^/Cl^−^ and K^+^ efflux (extracellular alkalization and acidification of the cytoplasm); activation of mitogen-activated protein kinase (MAPK); NADPH oxidase-induced production of oxidative species (reactive oxygen or nitrogen species); and, finally, the expression of defensive genes and feasible accumulation of secondary metabolites **Figure 10** (84).

The application of NPs to uberculat the plant ubercula system during stress conditions is an innovative improvement to the abiotic elicitors. As an example, one recent study utilized silver nanoparticles (AgNPs) for the improvement of plant biomass and essential polyphenol secondary metabolites in *C. uberculate* cell cultures (67). In this study, various doses of AgNPs and PGRs, either alone or in combination, were used for multiple productions of plant biomass and bioactive metabolites in *in vitro* callus cultures of *C. uberculate*. The results demonstrated that an optimal level of AgNPs, when synergistically used with PGRs (2,4-D and BA) in MS medium, substantially increased the callus proliferation, polyphenol concentrations, and the enzymatic antioxidant (SOD, POD, CAT, and Apx) activities. From this study, it may be concluded that the use of AgNPs as stress-induced elicitors can successfully enrich the bioactive compounds in callus culture of the critically endangered *C. uberculate*. Therefore, evaluation of the impact of NPs on cell growth and secondary metabolite biosynthesis pathways is crucial for a better understanding of the cellular mechanism underlying the plant–nanomaterial interaction.

**Figure 9:**
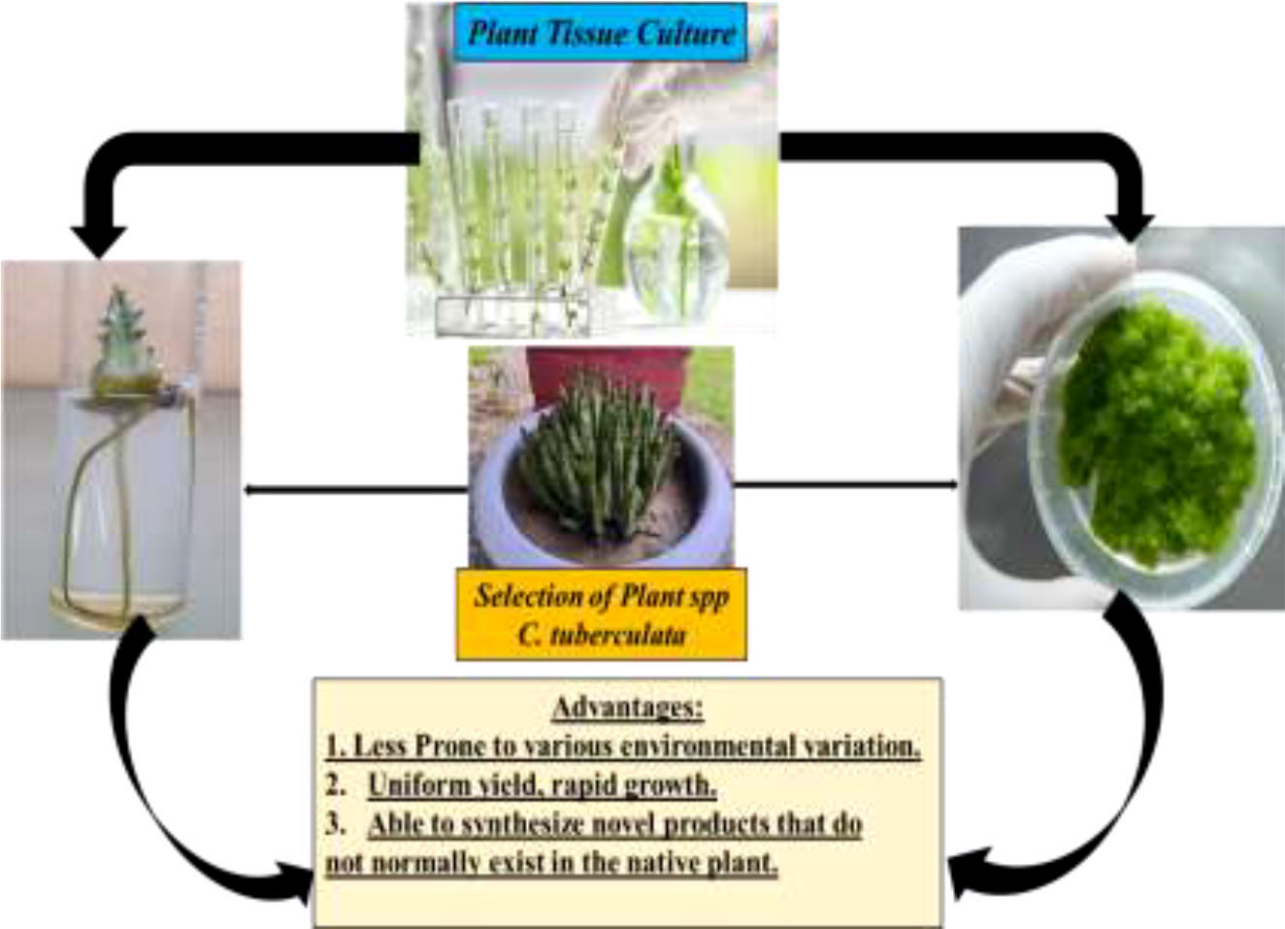
Advantages of plant cell culture technology.

**Figure 10:**
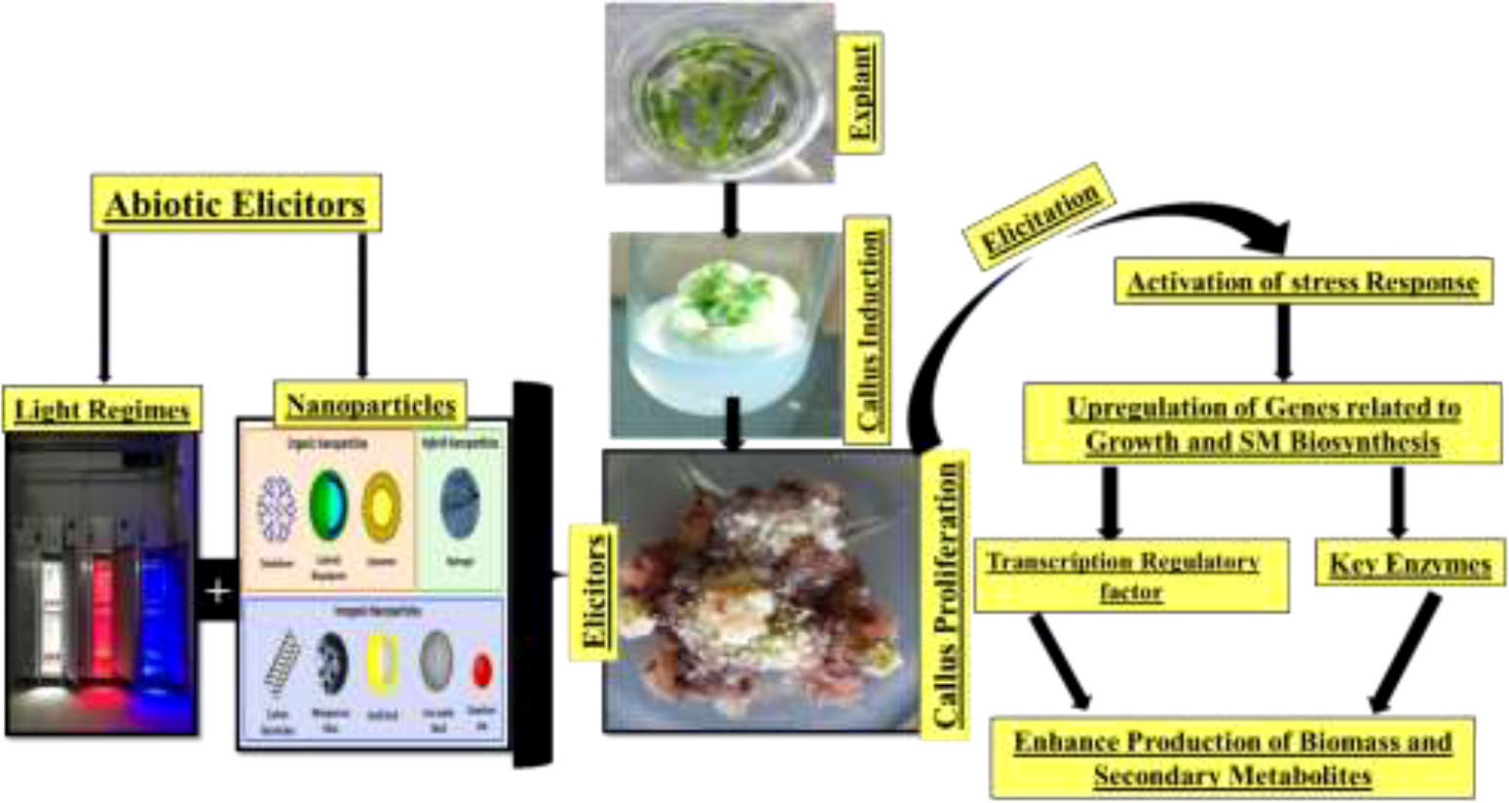
Abiotic elicitor nanoparticles regulate callus growth and antidiabetic secondary metabolite production in *Caralluma uberculate* callus.

**Figure 11:**
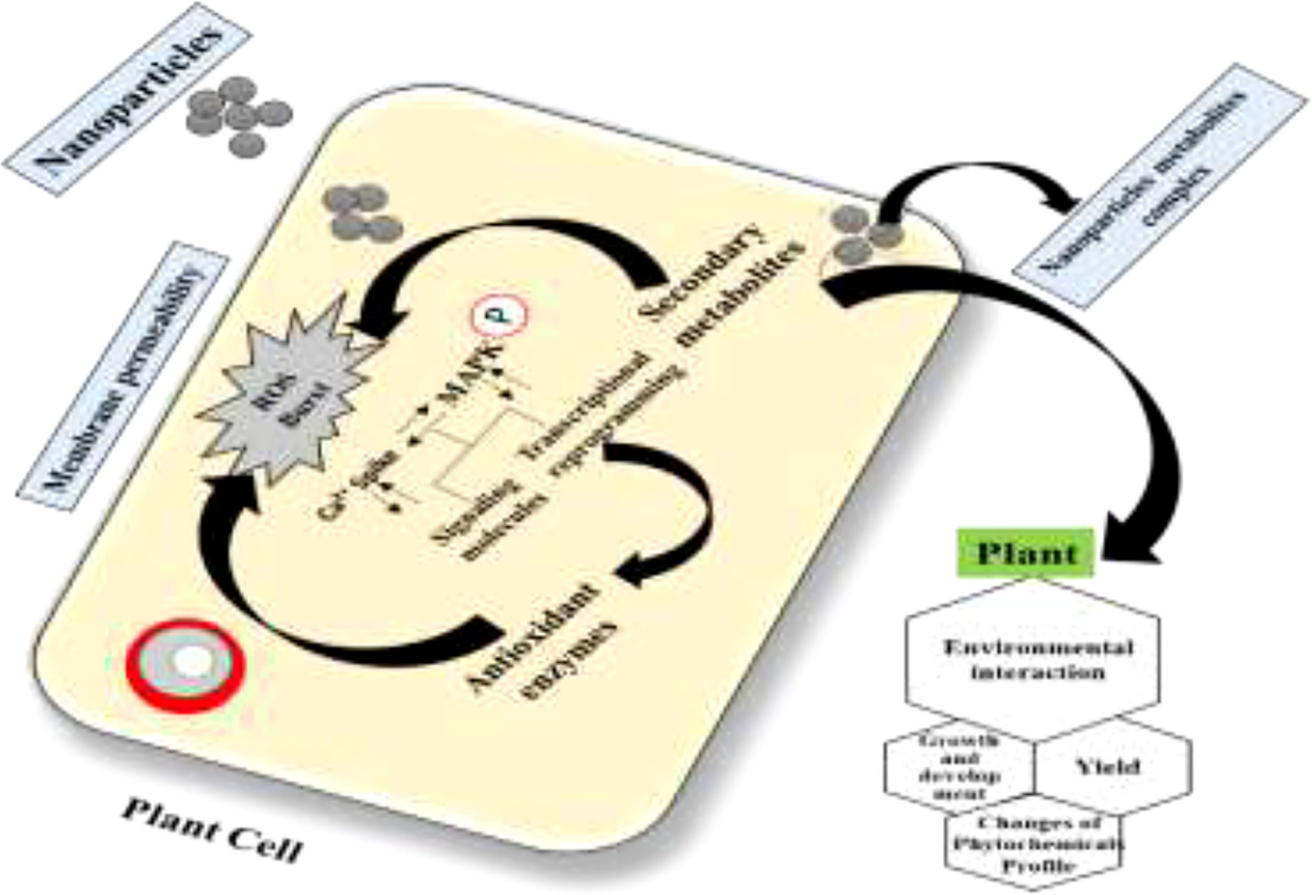
Proposed mechanism of action of nanoparticles in cell signaling and secondary metabolism in cell cultures through elucidation of the mitogen-activated protein kinase (MAPK) pathway (67).

However, we propose that NPs in the culture media might act as signaling elicitor compounds that affect the cell growth and the synthesis process of secondary metabolites. Exposure of plant cells to NPs ensues in oxidative outbursts and the production of ROS in the surrounding environment of plant cells. These oxidative species can damage the cell membrane. To survive in such a stressful environment, plants activate different mechanisms for scavenging ROS and stimulate their metabolomics process known as the MAPK route (**Figure 9**). Upon activation of the MAPK pathway, the plant polyphenol elements interact with ROS in a cascading fashion and scavenge them. Consequently, cell division is stimulated after the cellular response to achieve average plant cell’ growth and development with many pharmaceutically critical secondary metabolites (**Figures 10**, **11**) (67).

This section presented precise evaluation of the recent achievements concerning the use of nanomaterials in *in vitro* plant culture to improve the callus proliferation and metabolite production of *C. uberculate*. The need to incorporate more of the new-age nanomaterials as elicitors, such as zinc, iron, gold, and copper NPs, and the possibility of creating nanoenvironments for robust plant tissue cultures to conserve and improve biomass that could be a rich source of antidiabetic bioactive metabolites in the critically endangered *C. uberculate* species, are discussed in the prospects section.

## 6 Conclusion and future perspectives

Aside from the results presented on the medicinal value of *C. tuberculata*, this study will also attract the attention of clinicians and pharmacists regarding the design of antidiabetic drugs with the establishment of a non-toxic herbal medicine due to this plant being a rich source of volatile bioactive antidiabetic compounds. In light of the therapeutic potential of *C. tuberculata*, it could be used as an alternative to synthetic antidiabetic drugs. Previous studies reported that the phytochemicals of *C. tuberculata* showed prominent antidiabetic activity through various mechanisms, including inhibiting α-glucosidase and α-amylase and increasing insulin sensitivity and secretion. However, insufficient production in natural conditions does not meet the increasing pharmaceutical demand. Therefore, improving the production of this plant material is necessary. *In vitro* approaches such as callus culture and micropropagation through elicitation can be utilized for the conservation and enhancement of the plant biomass and the production of antidiabetic compounds at a considerably larger scale. Understanding the biosynthetic and regulatory pathways is essential to improving the agronomic and metabolic traits of *C. tuberculata.* Further research on these phytochemicals could uncover several targets for therapeutic interventions for DM. In addition, elucidation of the feasibility and the toxicity profile, despite the aforementioned advantages of plant-based products, is also a salient research concern.

## Author contributions

AA, Z-u-RM, and NR devised the study. AA wrote the first draft. Z-u-RM provided guidance and supervision. SM, AA, and IA edited and reviewed the manuscript. All authors contributed to the article and approved the submitted.
